# Endoscopic Radiofrequency Ablation Prolongs Survival of Patients with Unresectable Hilar Cholangiocellular Carcinoma - A Case-Control Study

**DOI:** 10.1038/s41598-019-50132-0

**Published:** 2019-09-23

**Authors:** Arne Bokemeyer, Philipp Matern, Dominik Bettenworth, Friederike Cordes, Tobias Max Nowacki, Hauke Heinzow, Iyad Kabar, Hartmut Schmidt, Hansjoerg Ullerich, Frank Lenze

**Affiliations:** 1 Department of Medicine B for Gastroenterology and Hepatology, University Hospital Muenster, Muenster, Germany; 2 Department of Internal Medicine I, Josephs-Hospital Warendorf, Warendorf, Germany

**Keywords:** Bile duct cancer, Bile ducts

## Abstract

The life expectancy of unresectable hilar cholangiocellular carcinomas (CCCs) is very limited and endoscopic radiofrequency ablation (ERFA) of the biliary tract may prolong survival. Our single-center-study retrospectively analysed all CCC cases, in whom ERFAs of the biliary tract were performed between 2012 and 2017 and compared these to historical control cases who received the standard treatment of sole stent application. ERFA was performed in 32 patients with malignant biliary strictures that were mainly caused by Bismuth III and IV hilar CCCs (66%). 14 of these patients received repeated ERFAs, for an overall performance of 54 ERFAs. Stents were applied after examination of all patients (100%). Adverse events occurred in 18.5% of examinations. Case-control analysis revealed that the survival time of cases with unresectable Bismuth type III and IV hilar CCCs (n = 20) treated with combined ERFA and stent application significantly increased compared to controls (n = 22) treated with sole stent application (342 +/− 57 vs. 221 +/− 26 days; p = 0.046). In conclusion, ERFA therapy significantly prolonged survival in patients with unresectable Bismuth type III and IV hilar CCC. As an effective and safe method, ERFA should be considered as a palliative treatment for all these patients.

## Introduction

Bile duct cancers around the hepatic fork are termed hilar cholangiocellular carcinomas (CCCs)^1^ and represent the most common type of all bile duct cancers at 70%^2,3^. Only 20–30% of CCC patients are diagnosed at a stage when surgical resection is feasible, and all others require palliative care comprising endoscopic stent placement for biliary drainage^1,4^. The survival time of unresectable hilar CCC Bismuth type III and IV, is alarmingly short and generally less than 1 year despite current available chemotherapy^5–7^.

Percutaneous radiofrequency ablation (RFA) therapy is an established treatment option for solid liver tumours and RFA is part of current guideline recommendations for the treatment of hepatocellular carcinomas^8^. RFA locally produces high temperatures, which lead to necrosis of the surrounding tissue resulting in reduction in tumour size and regrowth^9^. Endoscopic radiofrequency ablation (ERFA) catheters were first introduced less than 10 years ago, and these catheters are easily used with a standard-sized duodenoscope^10,11^.

Few ERFA studies with a limited number of patients have been published^12–21^ and only a minority of these reports suggest that ERFA can prolong the survival of patients with biliary strictures caused by pancreatic carcinomas and distal CCCs^13,17,20,21^. However, none of these studies included survival analyses of patients with Bismuth type III and IV CCCs despite the rather short life^5–7^.

Therefore, the present study generally assessed the efficacy and safety rates of ERFA therapy in malignant bile duct strictures and specifically evaluated the efficacy of ERFA procedures to improve survival rates in patients with unresectable Bismuth type III and IV CCCs compared to controls.

## Results

### Study population

We included 32 patients with malignant biliary strictures being treated with ERFAs. 14 of these patients (43.8%) received repeated ERFAs (n = 22) for a total of 54 ERFAs (Table 1). The patients were predominantly male (65.6%), and the mean age was 68 years (standard error of mean [SEM]: +/−11 years). CCC caused bile duct stricture in 75% of these cases of which 87.5% exhibited Bismuth type III and IV CCC. Other underlying causes for biliary strictures were pancreatic carcinomas (6.3%), gallbladder carcinomas (6.3%) and other metastatic diseases (12.5%). A total of 37.5% of the patients received palliative chemotherapy (Table 1). All patients died during the observation period.

**Table 1:** Characteristics of patients undergoing endoscopic radiofrequency ablation (ERFA) of the biliary tract in cases of malignant biliary strictures.

Variables	Patients (n = 32)
Age (in years)	68 (+/−11)
Female (%)	11 (34.4)
Male (%)	21 (65.6)
Number of ERFAs	54
- Initial ERFAs (%)	32 (59.3)
- Repeated ERFAs (%)	22 (40.7)
ERC-based ERFAs (%)	54 (100)
**Etiology of malignant bile duct strictures (%)**	
Cholangiocellular carcinoma	24 (75.0)
- Hilar cholangiocellular carcinoma	21 (87.5)
- IV°	19
- III°	2
- Distal cholangiocellular carcinoma	2 (8.3)
- Intrahepatic cholangiocellular carcinoma	1 (4.2)
Pancreas carcinoma	2 (6.3)
Gallbladder carcinoma	2 (6.3)
Other malignomas	4 (12.5)
Systemic palliative chemotherapy (%)	12 (37.5)

Arithmetic mean and standard error of mean (SEM) are reported for continuous variables, and frequencies and percentages are reported for categorical variables.

### Technical aspects of ERFA of malignant biliary strictures

Examinations combined with ERFAs of malignant biliary tract strictures were primarily performed due to a biliary obstruction without signs of cholangitis (66.7%) and less frequently due to signs of obstructive cholangitis (33.3%; Table 2). ERFA procedures were successfully completed in all cases, for a technical success rate of 100%. Eight watts and ten watts of power were applied during ERFA in most cases (40.8% and 35.3%, respectively; Table 2). Overlapping ERFA applications during one session were used in 62.9% of patients because of the spatial extent of the stricture. In detail, two overlapping ERFA applications were performed in 25 cases (46.3%), three applications were performed in 7 cases (13.0%), and more than three applications were performed in two cases (3.7%). An endoprosthesis was used after ERFA in all cases (100%): a plastic endoprosthesis was implanted in most cases (85.3%), and 14.8% of cases received self-expandable metal stents (SEMS; Table 2).

**Table 2:** Endoscopic radiofrequency ablation (ERFA) of malignant biliary strictures.

Variables	RFA (n = 54)
Indication for examination (%)	
Biliary obstruction without cholangitis	36 (66.7)
Biliary obstruction with cholangitis	18 (33.3)
Main localization of malignant bile duct obstruction (%)	
Hepatic fork	30 (55.6)
Common bile duct	15 (27.8)
Intrahepatic bile duct	4 (7.4)
Diffuse disease	5 (9.3)
Technical success of ERFA (per examination)	54 (100.0)
**Technical aspects of ERFA (per examination)**:	
Level of energy application	
8 Watts	22 (40.8)
10 Watts	19 (35.3)
Others	5 (9.3)
Overlapping ERFA applications	
No	12 (22.2)
Yes	34 (62.9)
Information not provided	8 (14.8)
Stent application following ERFA	54 (100)
Plastic endoprosthesis	46 (85.2)
Self-expandable metal stent (SEMS)	8 (14.8)

Frequencies and percentages are reported for categorical variables.

### Complications following ERFA of the biliary tract

No complications occurred in most cases (81.5%), but adverse events were observed in 18.5% of all examinations (Table 3). The most frequent complication was post-interventional cholangitis (11.1%; n = 6) followed by post-interventional pancreatitis (3.7%; n = 2). An intestinal perforation and pneumothorax concomitantly occurred in one patient (1.9%). The intestinal perforation was most likely a mechanical injury from the endoscope rather than the ERFA performance; the intestinal perforation as well as the pneumothorax could be treated conservatively. Cholecystitis was observed in another patient (1.9%; Table 3). One patient was transferred to the intensive care because of the above-mentioned intestinal perforation, but all other cases exhibited complications with a mild clinical course. No deaths occurred due to procedure-related side effects. The complication rate following ten watts ERFA application tended to be higher than the eight watts applications (46.2% vs. 9.1%; p = 0.07).

**Table 3:** Safety data following endoscopic radiofrequency ablations (ERFAs) of malignant biliary strictures (n = 54).

Variables	RFAs (n = 54)
Adverse events (in % per examination)	10/54 (18.5)
Cholangitis	6/54 (11.1)
Mild	2/54 (3.7)
Moderate	4/54 (7.4)
Severe	0/54 (0.0)
Pancreatitis	2/54 (3.7)
Mild	1/54 (1.9)
Moderate	1/54 (1.9)
Severe	0/30 (0.0)
Others	2/54 (3.7)
Median suspected prolonged hospital stays in case of adverse events (in d)	6

Percentages are reported for categorical variables.

### Survival of patients with Bismuth type III and IV hilar CCCs following ERFA: a case-control analysis

A case-control analysis was performed to compare ERFA+ endoscopic stent therapy to controls who only received endoscopic stenting and determine the significance of ERFA for the survival of patients with unresectable (Bismuth III and IV) hilar CCCs. 21 patients with Bismuth III and IV hilar CCCs were identified: in 20 of these patients the overall survival time was successfully determined and only one patient was lost to follow up. Finally, 20 patients with Bismuth III and IV hilar CCCs were included in the ERFA+ stent group (Table 4). The same inclusion criteria were used to select the control group. Out of 116 patients with a CCC, 22 with Bismuth type III and IV hilar CCC were identified who were treated at our hospital before ERFA was available and who exhibited a minimal survival of 45 d. Both groups were well matched and did not differ in important criteria such as the extent of disease (p = 0.607), mean age (p = 0.537), frequency and type of stent application (p = 1.000 and p = 0.656) and frequency of chemotherapy application (p = 0.899; Table 4).

**Table 4:** Case-control analysis of patient’ survival with unresectable (Bismuth type III and IV)-hilar cholangiocellular carcinomas undergoing endoscopic radiofrequency ablation (ERFA).

	Unresectable (Bismuth type III and IV)-hilar cholangiocellular carcinomas		p-value
	Patients with ERFA	Patients without ERFA	
Overall survival (in d)	342 (+/−57)	221 (+/−26)	0.046*
**Comparison of groups**:			
Klatskin-Tumor (Bismuth)	20	22	0.607
IV	19	20	
III	1	2	
Age (in years)	68 (+/−2)	66 (+/−)	0.537
Stent application following ERC (%)	20 (100.0)	22 (100.0)	1.000
Plastic endoprosthesis	17 (85.0)	20 (90.9)	0.656
Bare metal stent	3 (15.0)	2 (9.1)	
Palliative chemotherapy (%)	6 (30.0)	7 (31.8)	0.899
Gemcitabine+ Cis-/Oxaliplatin	3 (50.0)	3 (42.9)	
Gemcitabine	3 (50.0)	1 (14.3)	
Gemcitabine + Sorafenib	0 (0.0)	1 (14.3)	
Folfirinox	0 (0.0)	1 (14.3)	
Sorafenib	0 (0.0)	1 (14.3)	

Arithmetic means and standard error of mean (SEM) are reported for continuous variables, and percentages are reported for categorical variables. Statistical analysis was performed using Chi squared analysis and Mann-Whitney rank sum analysis. Patient with an additional surgical intervention for tumour resection and patients undergoing radiation or photodynamic therapy were excluded in both groups.

**Figure 1:**
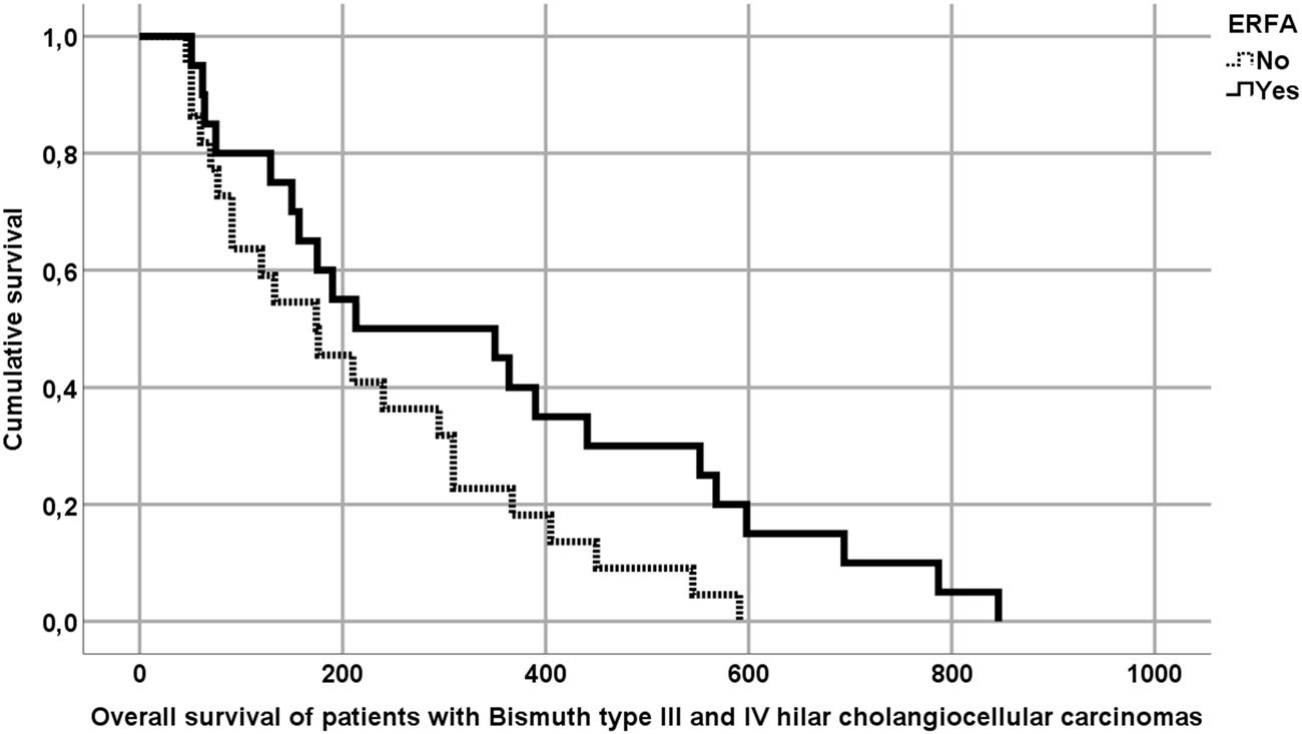
Case-control analysis of patient’ survival with Bismuth type III and IV hilar cholangiocellular carcinomas undergoing endoscopic radiofrequency ablation (ERFA). The survival of patients with a Klatskin-Tumor Bismuth III/IV undergoing ERFA+ endoscopic stent therapy was significantly longer than controls who solely received endoscopic stent therapy (342 d vs. 221 d; p = 0.046). Statistical analysis was performed using Kaplan-Meier-survival analysis.

Finally, Kaplan-Meier-analysis revealed that patients with unresectable Bismuth type III and IV CCCs treated with endoscopic stent placement + ERFA exhibited significantly prolonged survival compared to controls who received sole endoscopic stent therapy (342 d [SEM: +/−57] vs. 221 d [+/−26]; p = 0.046; Table 4 and Fig. 1).

## Discussion

To the best of our knowledge, this report is the first study to demonstrate that ERFA is an efficient and safe tool for the treatment of patients with unresectable Bismuth type III and IV hilar CCCs and significantly prolonged survival time compared to controls receiving standard treatment (342 d vs. 221 d; p = 0.046).

Steel *et al*. (2011) first introduced ERFA procedures for the treatment of malignant biliary obstruction^10^. Since then, several studies concerning ERFA procedures of malignant biliary strictures have been published. However, data are limited, and reports comparing the beneficial effects of ERFA therapy for the survival of patients with biliary cancer are rare^12–21^. Only a minority of studies included more than 50 ERFA procedures^12,13,20,21^. Comparing, our data set with 54 ERFA procedures belongs to the largest one’s currently available. A meta-analysis was published in 2016 summarizing parts of the above-mentioned ERFA studies including 263 patients: according to this analysis, the pooled technical success rate of ERFA-procedures was 96.8%^22^. Comparable, our technical success rate was 100%, which supports, that ERFA procedures are easily performed in most patients and reflects the technical expertise of the local investigators. Previous studies reported adverse events of 4–60%^14,17^ and the above-mentioned meta-analysis of Zheng *et al*. found a pool adverse event rate of 17% per procedure^22^. Our study cohort found a comparable complication rate of 18.5%. Of these, no complication lead to death and no patient required surgery. No previous report in humans analysed the onset of adverse events based on the level of applied ERFA power. Notably, the complication rate in our cohort tended to be higher in patients who received ten watts of ERFA compared to patients with eight watts of power (46% vs. 9%; p = 0.07). Whether higher energy improves biliary tract patency is unknown. However, experimental reports in swine demonstrate that the ablation area was larger and deeper with increased power^23^, and that higher power may induce greater thermal injury to adjacent tissues, why it might be speculated that higher applied energy might only be used in cases with an adequate parenchymal coverage^24^. Future studies should evaluate the energy level that provides the best balance between effective ERFA therapy and an acceptable rate of adverse events.

The survival time of patients with hilar CCCs, especially patients with unresectable Bismuth type III and IV hilar CCCs, is particularly short^5–7^. The median survival time of patients with unresectable perihilar CCC was 7 months in a recently published American registry analysis, and a Japanese study of patients with unresectable Bismuth type III/IV hilar CCCs published a median survival of 9 months^5,6^. We found a mean survival of 7.4 months for patients with unresectable Bismuth type III and IV hilar CCC in whom no ERFA was performed, which is comparable to previous reports.

Only four studies analysed the influence of ERFA procedures on the survival of patients with malignant biliary stricture compared to controls^13,17,20,21^. Kallis *et al*. published a retrospective case-control-analysis of 23 patients with unresectable pancreatic carcinomas that caused a malignant biliary obstruction^17^. These patients underwent ERFA in combination with SEMS, and were compared to 46 control cases who received only SEMS. The median survival of the ERFA group was significantly higher than the control group (226 d vs. 124 d; p = 0.01). These results suggest that ERFA can prolong the survival of patients with malignant biliary strictures; however, this study only included patients with pancreatic carcinomas^17^. Sharaiha *et al*. retrospectively analysed the effect of ERFA procedures on the survival of patients with a wide range of different biliary stricture aetiologies, including CCCs, pancreatic carcinomas, gallbladder carcinomas, gastric cancers and liver metastases of colon cancer^21^. Elucidating the survival data of 69 patients who underwent combined ERFA-procedures and stent placement compared to a matched cohort that was treated by endoscopic stent placement only, the authors found a significantly improved survival in patients with pancreatic cancer (5.9 vs. 14.6 months; p < 0.001) and CCCs (6.2 vs. 17.7 months; p < 0.001). Limiting, this study included biliary strictures caused by different malignomas, and the authors did not specify their findings to distinct types of CCCs (hilar CCCs vs. distal CCCs)^21^. Another retrospective case-control study analysed the effect of metal stenting with or without ERFA for unresectable CCCs and exclusively included patients with Bismuth type I hilar CCCs and distal CCCs^13^. Their case-control analysis revealed that patients who received ERFA+ SEMS application (n = 34) exhibited significantly longer survival than SEMS application alone (n = 42; p = 0.036)^13^. The first randomized controlled trial to analyse the survival of patients with an extrahepatic CCC following ERFA therapy was recently published: Yang *et al*. included 65 patients with unresectable extrahepatic CCCs (except Bismuth type III/IV hilar CCCs), and randomized these patients to ERFA in combination with biliary stenting (n = 32) or biliary stenting alone (n = 33). The survival time was significantly longer in the ERFA+ stent group than the stent-only group (13.2 vs. 8.3 months; p < 0.001). In summary, only three previous studies analysed the effect of ERFA therapy on the survival of CCC patients^13,20,21^, but none of these studies included patients with unresectable Bismuth type III and IV hilar CCCs, who especially suffer severe disease progression and short survival.

Our case-control analysis addressed this current lack of data. We exclusively included cases with unresectable Bismuth type III and IV hilar CCCs (n = 20) who received standard endoscopic care including stent placement +ERFA procedures and compared these cases to a well-matched historic cohort of patients (n = 22) who received endoscopic stent placement only. Our study is the first report to demonstrate that patients with unresectable Bismuth type III and IV hilar CCCs following ERFA therapy + stent application significantly prolonged survival by greater than 100 d compared to matched controls who received endoscopic stent therapy alone (342 d vs. 221 d; p = 0.046).

Long time, photodynamic therapy of the biliary tract was believed to significantly prolong survival in patients with unresectable CCCs^25,26^. However, a recent prospective multicentre trial found a worse outcome for patients treated with photodynamic therapy in combination with endoscopic stent placement compared to sole stent treatment^27^. Next to these conflicting results, photodynamic therapy requires a prior injection of an intravenous photosensitizer, which makes patients highly vulnerable to sunlight and potentially affects patients’ quality of life. By contrast, ERFA is not accompanied by these systemic side effects and may represent a more favourable treatment for advanced hilar CCC.

Our study has several limitations. First, we report data from a single-centre study, and our final data set consisted of only 54 ERFA therapies performed in 32 patients; however, it represents one of the largest data sets of ERFA therapies applied to malignant biliary strictures available to date. Second, this study is retrospective and for the case-control-analysis, controls were chosen from patients before ERFA was available at our hospital; however, we collected a homogenous and complete data set featuring detailed clinical and endoscopic reports and historic controls were selected out of an equal time period of six years directly before ERFA was available, why endoscopic standard technique including stent placement was most likely similar effective to the endoscopic standard treatment at the time patients received ERFA-therapy. Third, the prescribed inclusion criteria for our case-control analysis may have created a systematic sampling error and propensity score matching might be able to reduce bias; however, the matching algorithm would inherently reduce the already limited number of included patients. To address this limitation, we used the same, detailed and well-defined inclusion criteria for the selection of both groups (ERFA+ stent-group vs. stent-only-group) and could furthermore show, that both groups were equally distributed in important criteria known to influence survival like the age of the patients, the extent of disease, the use of endoprostheses and the application of systemic palliative chemotherapy. Fourth, only 32 of 40 patients undergoing ERFA at our hospital were included in whom ERFA of the biliary tract, and not of a pre-inserted SEMS, was performed; however, due to the exclusion of cases with a primary ERFA of a SEMS, we established a comparable, homogeneous ERFA group avoiding data mixture. Fifth, the administration of systemic palliative chemotherapy can prolong survival of patients with an unresectable hilar CCC^28,29^. We cannot exclude, that the slightly different types of chemotherapeutics applicated to the patients of our case-control analysis might influence overall survival; however, only the minority of our patients received chemotherapy at all, the rate of patients receiving chemotherapy was equal in both groups and furthermore, the types of chemotherapeutics applicated were generally comparable.

Taken together, the survival of patients with a hilar CCC is still limited despite the application of current available chemotherapies. Our data suggest, that ERFA is a valuable, less invasive and easy-to-use tool to substantially prolong the overall survival of patients with unresectable Bismuth type III and IV hilar CCC. However, further studies are needed to strengthen these findings.

## Methods

### Study design and inclusion criteria

This retrospective study was performed at the Department of Medicine B for Gastroenterology and Hepatology of the University Hospital Muenster, Germany. The Ethics Board of the Westphalian Wilhelms-University of Muenster and the Medical Council of Westphalia-Lippe, Germany approved the study, which conformed to the ethical guidelines of the 1975 Declaration of Helsinki. As approved by the Ethics Board, informed patient consent was not required for this study because of its retrospective design. To reduce known sources of bias, this study was reported according to the checklist of the STROBE statement, wherever applicable and appropiate^30^.

Data from all patients ≥18 years of age who underwent an ERFA of the biliary tract using the *Habib*^*TM*^ *EndoHPB Biopolar Radiofrequency Catheter* (Boston Scientific, Marlborough, USA) between a six-year period from January 2012 to December 2017 were retrieved from the clinical data system. Of these, cases were only included, if the patients had (i) an unresectable malignoma of the biliary tract, and (ii) the ERFA was not primarily performed on a pre-implanted SEMS. SEMS insertion was allowed after the performance of the first ERFA over time.

The primary outcome of this study was to assess the efficacy of ERFA procedure to improve survival rates in patients with unresectable Bismuth type III and IV CCCs compared to controls. The secondary outcome of this study was to evaluate the safety of ERFA procedure for the treatment malignant biliary strictures in general.

For the case-control analysis with patients with an unresectable Bismuth type III and IV hilar CCC, only ERFA-cases were included in whom all following additional criteria were met: (i) patients had a Bismuth type III or IV hilar CCC; (ii) an endoscopic intervention including stent placement to achieve biliary drainage was performed; and (iii) the survival time of the patient could be determined. Exclusion criteria were defined as (i) the performance of other local ablative therapies including a photodynamic therapy of the biliary tract and (ii) the surgical resection of the tumor. Patients receiving palliative chemotherapy were not excluded from this study because this treatment is a part of the currently established standard of care.

Control cases were carefully selected from our hospital information system from all patients ≥18 years of age who suffered CCC between an equal six-year time period from January 2006 to December 2011. This time interval was chosen, because the ERFA technique was not available at our tertiary referral centre during this time. Control cases without ERFA procedures needed to fulfill all the above enlisted criteria plus a minimal survival of at least 45 d after initial diagnosis.

To reduce bias, cases and controls were carefully selected by two authors (A.B. and P.M.) following the above enlisted detailed inclusion criteria. Furthermore, to minimize confounders, the case and the control group were compared for equality of important criteria which are known to influence survival like the age of the patients, the extent of disease, the use of endoprostheses and the application of systemic palliative chemotherapy.

### Technical aspects of ERFA of the biliary tract

Highly experienced endoscopists performed all examinations according to generally accepted guidelines using an endoscopy retrograde cholangiography (ERC) case volume greater than 200/year^31,32^. All patients received (prophylactic) antibiotic treatment. The *Habib*^*TM*^ *EndoHPB Biopolar Radiofrequency Catheter* (Boston Scientific, Marlborough, USA) was advanced into the biliary tree under fluoroscopic guidance until it was within the malignant stricture^11^. The catheter was 180 cm long, with a diameter of 2.7 mm, and it is easily inserted through a 3.2 mm working channel of a standard duodenoscope^11^. Radiofrequency power was delivered using an ERFA generator (ERBE ICC 200) for 90 seconds. A resting period of 30 seconds was used after ERFA, before the catheter was removed. Overlapping ERFA applications were performed depending on the length of the biliary stricture. The technical success of ERFA procedures was defined as the complete performance of the ERFA procedure: this included at least one full episode of radiofrequency application; the choice to perform multiple overlapping ERFA applications during one session was up to the operator.

### Safety analysis

The following complications after ERFA were documented: (i) post-interventional pancreatitis was diagnosed if the onset of abdominal pain was accompanied by a three-fold increase in serum lipase levels within 48 hours of the examination; (ii) post-interventional cholangitis was defined as the onset of fever and newly or significantly higher inflammatory markers that required antibiotics within three d of the examination; both, pancreatitis and cholangitis were graded as mild, moderate and severe cases depending on the additionally required length of hospitalization based on a recent guideline^33^ (mild = 1–3 d, moderate = 4–10 d and severe = >10 d of additional hospitalization); (iii) severe bleeding was diagnosed if bleeding during an intervention was observed that required immediate endoscopic therapy or if a drop in haemoglobin of two points or more was observed and iv) any other relevant procedure-related complication was documented.

### Statistical analysis

The data analysis was performed using IBM SPSS Statistics 25.0 (IBM Corp., Armonk, USA). The contingency table-derived data were calculated using StatPages^34^. Arithmetic mean and standard error of mean (SEM) are reported for continuous variables, and frequencies and percentages are reported for categorical variables.

## Data Availability

All data generated or analysed during this study are included in this article. The orginal datasets are available from the corresponding author on reasonable request.
